# Incidence and characteristics of pediatric patients with Crohn's disease undergoing surgery: A cross‐sectional study

**DOI:** 10.1002/jpr3.70052

**Published:** 2025-06-24

**Authors:** Hugo Gagnon, Marie‐Frédérique Paré, Guillermo Costaguta, Marie‐Catherine Turcotte, Prévost Jantchou, Laurence Chapuy, Colette Deslandres

**Affiliations:** ^1^ Centre Hospitalier Universitaire Sainte‐Justine, Division of Gastroenterology, Hepatology and Nutrition Montréal Quebec Canada; ^2^ Centre de Recherche du CHU Sainte‐Justine Université de Montréal Montréal Quebec Canada

**Keywords:** biologics, IBD, incidence, surgery, treatments

## Abstract

**Objectives:**

Despite biological treatments reducing the burden of pediatric inflammatory bowel disease, many patients still require surgery. Data on pediatric patient characteristics and surgical incidence are limited, often based on adult studies. This study aimed to assess the characteristics of pediatric Crohn's disease (CD) at diagnosis and compare surgery rates between two periods (before and after 2019) to understand which patients require surgery.

**Methods:**

We analyzed pediatric CD patients who underwent surgery at CHU Sainte‐Justine, Montreal, between 2014 and 2023. Descriptive statistics and the Mann–Whitney *U*‐test were used to compare means, while Kaplan–Meier curves assessed surgery‐free survival, with significance set at *p* < 0.05.

**Results:**

The overall surgery incidence was 5.2/1000 person‐years. Surgery rates were lower for patients diagnosed after 2019 than before 2019 (5.6/1000 vs. 14.7/1000 person‐years). There were no significant differences in age at diagnosis, CD Paris score, reason for surgery, or disease severity. Among CD patients, surgeries were more frequent before 2019 (11.5% vs. 2.8%, *p* < 0.001). The reduction in surgery rates since 2019 is likely due to earlier initiation of biologics, with a median initiation of 14 days after 2019 compared to 142 days before 2019 (*p* = 0.01).

**Conclusion:**

The reduced incidence of surgery in pediatric CD is a significant achievement. Increased use of infliximab, proactive drug monitoring, and better nonresponder management likely contribute to this improvement.

## Abstract (highlights)

**What is Known:**

- The prevalence of Crohn's disease (CD) is constantly increasing, particularly in Canada.
- Despite the addition of multiple biologics and small molecules, surgery remains a necessary treatment for selected patients.

**What is New:**

- This is one of the recent North American studies to establish the incidence of CD surgery in a tertiary pediatric center (5.2/1000 person‐years).
- This study supports the idea that the rapid initiation of biologics, along with proper treatment optimization, can have an impact on reducing the surgery rate in pediatric CD.

## 1 INTRODUCTION

The prevalence of inflammatory bowel disease (IBD) has significantly increased in recent years. In Canada, the current prevalence is estimated to be around 825 per 100,000 and is projected to reach 1.1% by 2035.^1^ Recently, medical treatments have increasingly shifted towards biologics, including antitumor necrosis factor agents, anti‐integrins, and anti‐interleukin therapies, as well as small molecules such as Janus kinase inhibitors. These therapies have shown better responses in the management of Crohn's disease (CD) and ulcerative colitis (UC) compared to previous treatments such as thiopurines and methotrexate.^2^, ^3^, ^4^ In the last 10 years, multiple studies have looked more closely into the early and rapid use of biologics and have shown benefits in long‐term outcomes.^5^, ^6^, ^7^, ^8^

According to the latest North American Society for Pediatric Gastroenterology, Hepatology and Nutrition (NASPGHAN) guideline on postoperative recurrence, the primary reasons for surgery in CD patients are treatment failure and poor growth, with the most common procedure being ileocecal resection.^9^

Although the increasing use of biological treatments has helped reduce the disease burden, surgery remains necessary for many patients. According to a study by Zhao et al.,^10^ the surgery rate for adults with CD is still 10‐30%, and for those with UC, approximately 5%–10% within 5 years. The CAROUSEL study by Chaparro et al.^11^ indicated that pediatric IBD is not associated with a higher risk of surgery than adults and that the median time from diagnosis to surgery is generally longer in children (15 months) than in adults (7 months). To our knowledge, only one recent study by Lujan et al.^8^ indicates a modest risk reduction of surgery for CD associated with the very early initiation of biologics.

Given the limited data currently available on surgical rates in the era of biologics for pediatric patients, this study aimed to assess the incidence of surgery in a pediatric CD population, the characteristics of those patients at diagnosis and to compare the rate of surgeries during follow‐up between two periods: before 2019 (January 1, 2014, to December 31, 2018) and after 2019 (January 1, 2019, to December 31, 2023).

## 2 METHODS

### 2.1 Ethics statement

This study obtained CHU Sainte‐Justine institutional review board approval (IRB #2024‐6816) on August 27, 2024.

### 2.2 Study design

This was a single‐center retrospective study. The electronic medical files of all pediatric patients with CD who underwent surgery between January 1, 2014, and December 31, 2023, at the CHU Sainte‐Justine, a tertiary pediatric center in Montreal, were reviewed. This Canadian reference center is one of the largest pediatric IBD centers, with more than 160 new IBD cases diagnosed each year.

### 2.3 Population

Inclusion criteria were patients aged 0–18 years old, diagnosed with CD at CHU Sainte‐Justine and identified in our local PediData IBD database,^12^ who underwent surgery for intestinal resection. Medical files were reviewed to evaluate whether the surgery had occurred within the timeframe. Each file corresponding to these criteria was retained. Exclusion criteria were patients with UC, as the number of colectomies was low, and the patients who underwent surgery solely for perianal disease without intestinal resection.

### 2.4 Data collection

The data collection began in 2014, when the CHU Sainte‐Justine implemented its electronic medical records system, which enabled the creation of the IBD database we are currently using. Although access to biological treatment dates back to the early 2000s, it wasn't until 2019 that therapeutic drug monitoring (TDM) was used uniformly between attendings at CHU Sainte‐Justine, therefore explaining the timeframe for each period. At that time, the attending gastroenterologists at CHU Sainte‐Justine started to systemically measure and standardize infliximab trough levels, aiming for a minimum of 15 µg/mL before the third dose and 10 µg/mL before the fourth dose of infliximab. Therefore, patients were separated into two periods: the patients diagnosed before 2019 (January 1, 2014, to December 31, 2018) and the ones diagnosed after 2019 (January 1, 2019, to December 31, 2023), as the management of biologics changed in 2019.

The following demographic variables were collected from medical files: sex, age at diagnosis, CD Paris classification, and Pediatric Crohn's Disease Activity Index (PCDAI) at diagnosis. For the surgery variables, date of surgery, age at surgery, reason for surgery, preoperative fecal calprotectin level, number of medications, and types of medications were collected.

To calculate the surgery annual incidence, we included the number of patients under 19 years of age with a diagnosis of IBD already made before 2014, as well as the number of new diagnoses made between 2014 and 2023 with the PediData IBD database.^12^

### 2.5 Statistical analysis

In this retrospective study, descriptive statistics were performed with Prism version 9.5.1. The survival calculation was performed using Kaplan–Meier curves and the log‐rank test. For continuous variables, means and standard deviations were used for normally distributed data or medians (range) and interquartile range (Q1:Q3) for non‐normal distribution; for categorical variables, frequency distributions and Fisher's exact test were used. To compare continuous variables between subgroups, the Mann–Whitney *U*‐test was used. The results were considered significant with a two‐tailed *p* < 0.05 and a confidence interval of 95%.

## 3 RESULTS

Between 2014 and 2023, 57 pediatric CD patients underwent surgery. During the first period before 2019, 42 patients were operated on, and 15 after 2019.

### 3.1 Incidence

**Figure 1:**
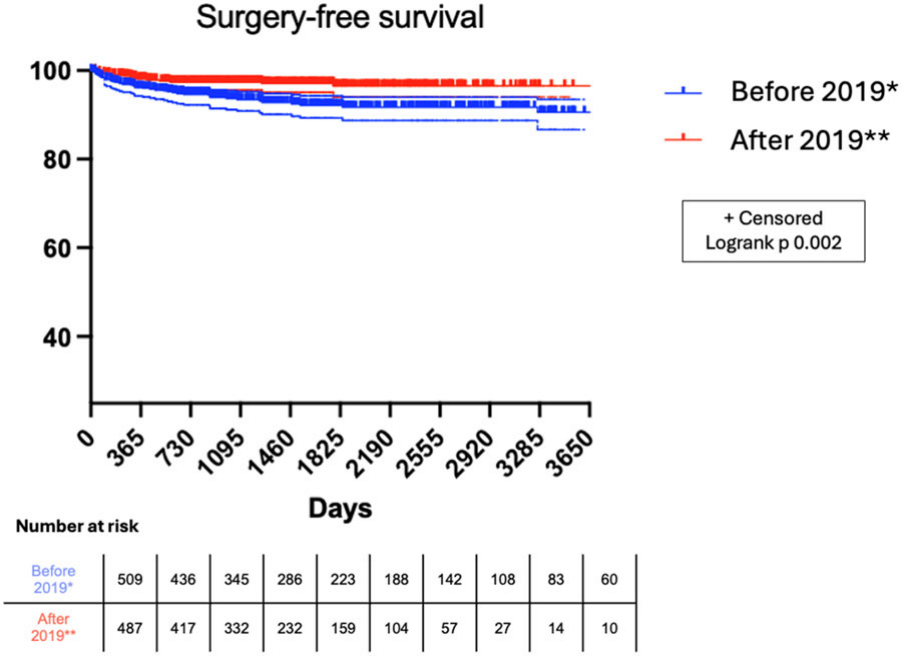
Surgery‐free survival according to diagnosis period. *January 1, 2014, to December 31, 2018. **January 1, 2019, to December 31, 2023.

Between 2014 and 2023, 899 new pediatric CD cases were diagnosed at our institution; of these, 366 were diagnosed in the first period and 533 in the second period. Two hundred and six patients who had been diagnosed before the study period were still under pediatric care (less than 19 years of age) and thus were considered in the calculation of incidence. During the study period, 57 patients underwent surgery with a global surgery rate of 5.2%. The general incidence of surgery was 5.2/1000 person‐years, with a significant decrease during the second period compared to the first period, from 14.7/1000 person‐years to 5.6/1000 person‐years (Figure 1, *p* = 0.002). This difference stems mainly from the marked decrease in surgery with 42 (7.3%) in the first period and 15 (2.8%) in the second period (*p* < 0.001) (Table 1).

**Table 1:** Patients requiring surgery in a pediatric Crohn's Disease (CD) cohort.

	Number of patients having surgery	Total number of patients in the cohort	%	*p* value
Diagnosis before 2019*	42	366	11.5	**<0.0001**
Diagnosis after 2019**	15	533	2.8	

*Note*: The bold values are indicate statistically significant p < 0.05.

* January 1, 2014, to December 31, 2018.

** January 1, 2019, to December 31, 2023.

### 3.2 Reasons for surgery

Fifty‐seven patients underwent surgery: 13 for refractory disease (22.8%), 10 for persistent abscess (17.5%), 28 for a stenotic sub‐occlusion that developed during the disease course (49.1%), 5 for the resection of a stenotic segment present at diagnosis (8.8%), and 1 patient for failure to thrive (1.8%) (Table 3). Although no statistical differences were found in the reasons for surgery between the periods, a numerical difference was noted, as 60% of the patients in the second period underwent surgery due to bowel stenosis development versus 45.2% in the first period (*p* = 0.34). It should also be noted that no patient in the second period required surgery due to stenosis that was present at diagnosis, although this difference was not significant.

### 3.3 Age and sex

There was no difference in the sex of patients undergoing surgery, with 45.2% and 46.7% of the patients being male in the first and second periods, respectively (Table 2). The median (interquartile) age at diagnosis in the whole cohort was 14.1 years (Q1:Q3 = 12.0:15.2), and there was no statistically significant difference between the periods (14.0 vs. 14.8 years, *p* = 0.14). Similar findings were noted regarding the age at the time of surgery, with a median age of 15.2 years (Q1:Q3 = 14.0:17.1) and a median of 15.0 and 15.4 years for the first and second periods, respectively (*p* = 0.19). The median time between diagnosis and surgery was 10.5 months, and there was no statistical difference between the first period (10.6 months, Q1:Q3 = 3.1:29.7) and the second period (11.3 months, Q1:Q3 = 3.4:16.7, *p* = 0.64).

**Table 2:** Patient characteristics.

	Diagnosis before 2019* (*N* = 42)	Diagnosis after 2019** (*N* = 15)	*p* value
	*n* (%)	*n* (%)	
Sex			
Female	23 (54.7)	8 (53.3)	0.93
Male	19 (45.2)	7 (46.7)	
CD Paris A			
A1a	9 (21.4)	0 (0)	**0.02**
A1b	30 (71.4)	15 (100)	
A2	3 (7.1)	0 (0)	
CD Paris B			
B1	19 (45.2)	6 (40.0)	0.73
B2	14 (33.3)	6 (40.0)	0.65
B3	9 (21.4)	3 (20.0)	0.91
CD Paris L			
L1	24 (57.1)	10 (66.7)	0.53
L2	2 (4.8)	1 (6.7)	0.78
L3	16 (38.1)	4 (26.7)	0.44
CD Paris L4			
L4a	11 (26.2)	5 (33.3)	0.61
L4b	8 (19.0)	1 (6.7)	0.27
L4aL4b	4 (9.5)	2 (13.3)	0.69
No	19 (45.2)	7 (46.7)	0.93
Perianal involvement			
Yes	7 (16.7)	2 (13.3)	0.77
No	35 (83.3)	9 (86.7)	
Age at diagnosis, median (Q1:Q3) (years)	14.0 (11.5:15.0)	14.8 (12.4:15.9)	0.14
Age at surgery, median (Q1:Q3) (years)	15.0 (13.6:17.1)	15.4 (15.1:17.1)	0.19
Time to surgery, median (Q1:Q3) (months)	10.6 (3.1:29.7)	11.3 (3.4:16.7)	0.64
PCDAI at diagnosis, median (Q1:Q3)	38.8 (30:50)	36.3 (30.1:42.5)	0.78

*Note*: The bold values are indicate statistically significant p < 0.05.

* January 1, 2014, to December 31, 2018.

** January 1, 2019, to December 31, 2023.

### 3.4 CD phenotypes

Of the 57 patients, 9 (15.8%) were A1a, 45 (78.9%) were A1b, and 3 (5.3%) were A2 at the time of diagnosis (Table 2). There was a significant difference in the age distribution between the periods, with 71.4% of patients in the first period being A1b versus 100% of those in the second period (*p* = 0.02). Regarding disease localization, the distribution of the entire cohort was 59.6% L1, 5.3% L2, and 35.1% L3. No significant differences were observed between the periods. Furthermore, upper gastrointestinal tract involvement was also similar for L4 phenotypes. Of the 57 patients, 16.7% had perianal involvement, with no significant difference between the periods analyzed (16.7% vs. 13.3%, *p* = 0.77). The disease phenotypes at diagnosis were as follows: 43.9% had a B1, 35.1% had a B2, and 21.1% had a B3; no difference was noted between the two periods.

### 3.5 Disease activity before surgery

The median PCDAI at the time of diagnosis was 37.5 (Q1:Q3 = 30:45), with those diagnosed during the first period having a median of 38.8 (Q1:Q3 = 30:50) versus a median of 36.3 (Q1:Q3 = 30.1:42.5) during the second period (*p* = 0.78) (Table 2). In the whole cohort, the median fecal calprotectin level was 1950 µg/g (Q1:Q3 = 689:2100) before surgery, with no difference between those from the first and second periods (median = 1950 µg/g, Q1:Q3 = 844:2100 vs. 1764 µg/g, Q1:Q3 = 329:2100) (Table 3).

**Table 3:** Surgical characteristics.

	Diagnosis before 2019* (*N* = 42)	Diagnosis after 2019** (*N* = 15)	*p* value
	*n* (%)	*n* (%)	
Reasons for surgery			
Abscess	7 (16.7)	3 (20.0)	0.77
Failure to thrive	1 (2.4)	0 (0.0)	0.56
Refractory disease	10 (23.8)	3 (20.0)	0.77
Stenosis/occlusion	19 (45.2)	9 (60.0)	0.34
Stenosis from diagnosis	5 (11.9)	0 (0.0)	0.17
Number of medications before surgery			
0	5 (11.9)	2 (13.3)	0.89
1	8 (19.0)	3 (20.0)	0.94
2	7 (16.7)	7 (46.7)	**0.01**
3	12 (28.6)	0 (0.0)	**0.02**
4	5 (11.9)	1 (6.7)	0.17
5	1 (2.4)	1 (6.7)	0.45
6	1 (2.4)	0 (0.0)	0.56
7	1 (2.4)	0 (0.0)	0.56
8	2 (4.8)	1 (6.7)	0.78
Medications before surgery			
5‐ASA	5 (11.9)	1 (6.7)	0.58
Adalimumab	8 (19.0)	4 (26.7)	0.54
Infliximab	29 (69.0)	8 (53.3)	0.28
Methotrexate	14 (33.3)	2 (13.3)	0.14
Steroids	25 (59.5)	10 (66.7)	0.63
Thiopurines	17 (40.5)	1 (6.7)	**0.02**
Tofacitinib	0 (0.0)	1 (6.7)	0.09
Upadacitinib	1 (2.4)	0 (0.0)	0.56
Ustekinumab	5 (11.9)	5 (33.3)	0.06
Vedolizumab	1 (2.4)	2 (13.3)	0.11
Preoperative Calprotectin levels, median (Q1:Q3) (µg/g)	1950 (689:2100)	1764 (329:2100)	0.52

	Diagnosis before 2019* (*N* = 35)	Diagnosis after 2019** (*N* = 14)	*p* value
Time from diagnosis to first biologics, median (Q1:Q3) (days)	142 (7:313)	14 (1:60.8)	**0.01**

*Note*: The bold values are indicate statistically significant p < 0.05.

* January 1, 2014, to December 31, 2018.

** January 1, 2019, to December 31, 2023.

### 3.6 Medications received before surgery

When analyzing the total number of medications patients received before surgery, 12.3% had none, 19.3% had received only one medication, 24.6% had received two, 21.1% had received three medications, 10.5% had four medications, and less than 6% received five, six, seven, or eight medications, respectively (Table 3). We did not find differences when comparing the mean number of medications received during each period (2.6 in the first and 2.3 in the second, *p* = 0.32). However, a significant difference was noted when analyzing each period individually. In the first period, 16.7% had received two medications and 28.6% had received three medications; while in the second period, 46.7% had received two medications (*p* = 0.01) and 0% had received three (*p* = 0.02). On the other hand, 76.2% of the patients in the first period had received three or fewer medications before surgery, similar to 66.7% of the patients in the second period, which was not significant (*p* = 0.48).

Regarding the type of medication used, 64.9% had received infliximab, 61.4% had received high‐dose steroids, 28.1% had received methotrexate, 31.6% had received thiopurines, 21.1% had received adalimumab, 10.5% had received mesalamine, 17.5% had received ustekinumab, 5.3% had received vedolizumab, and 1.8% each for tofacitinib and upadacitinib (Table 3). The distribution of these medications was similar between the periods, except for a significantly lesser use of thiopurines in the latest period (40.5% vs. 6.7%, *p* = 0.02).

Regarding the timing before using biologics, the difference between the delay to initiation was significant (*p* = 0.01). The median delay before starting biologics for the 2014‐2019 period was 142 days (Q1:Q3 = 7:313), with more than half the patients starting the treatment 3 months after diagnosis. For the 2019–2023 period, the median delay was 14 days (Q1:Q3 = 1:60.8), with only two patients starting the medication after 3 months, including one patient whose parents refused biologics for months before accepting despite multiple discussions. Subgroup analyses to compare early versus later onset of biologics were not performed, as virtually no patients had a later onset during the 2019–2023 period, and the groups would have been too small.

## 4 DISCUSSION

In this study, the incidence of surgery was 14.7/1000 person‐years for pediatric patients with CD with a diagnosis before 2019, and the incidence after 2019 was 5.6/1000 person‐years. To our knowledge, this is among a small number of North American studies that establish the incidence of surgery in a tertiary pediatric center and is an important statistic for newly diagnosed patients. One study by Jakobsen et al.^13^ also demonstrated a downward trend from 2007 to 2009 compared to 1998 to 2006, with an incidence of 4.2/100 person‐years, which was more than 10‐fold the current incidence in our center 15 years later.

Among patients, those requiring surgery were more frequent before 2019 compared to after 2019 (11.5% vs. 2.8% of newly diagnosed patients, *p* < 0.0001). A study by Adamiak et al.^14^ also showed a surgery rate of 17% at 4 years in newly diagnosed IBD, a number that seems to have improved in our most recent period.

Regarding patients' characteristics, the median age at diagnosis was 14.1 years, and the median age at surgery was 15.2 years, similar to other comparable studies.^15^ Contrary to the initial hypothesis that a potentially longer time between diagnosis and surgery was expected for patients operated on more recently (2019–2023), there was no significant difference. This could be explained by the fact that some of the patients who required surgery could represent a subgroup of more severely ill patients who would have inevitably undergone surgery, as in the NOD2 variants that have a higher risk of complicated disease and surgery.^16^ However, the significantly faster initiation of biologics in 2019–2023 with virtually all patients in this group having an early initiation (<3 months) could explain the smaller number of patients needing surgery in this period, as several studies have shown the efficacy of this early initiation of biologics in the 3 months following diagnosis.^5^, ^6^, ^7^, ^8^, ^17^

CD phenotypes were mostly teenagers with a non‐stricturing or non‐penetrating disease with an ileocecal disease (A1b, B1, L1). Perianal involvement was observed in a minority of patients (15.8%). A1b and perianal involvement proportions were similar to other studies defining phenotypes at diagnosis, and most of our cohort with perianal involvement was also male.^18^, ^19^ When looking at the CHU Sainte‐Justine IBD cohort in a previous study, 28% of patients were classified as L1 and 84.8% as B1, which is comparable to other studies that describe 16.9%–20% of L1 and 87% of B1 patients.^13^, ^18^ However, when analyzing this surgery cohort, fewer patients were B1 and more than usually reported were L1, which is most likely explained by a selection bias of patients with a severe phenotype requiring surgery. As for sex‐based disparity, males were previously reported to be more prone to require surgery; however, this was not the case in this study.^20^ This could be correlated to the fact that we observed more relapses and lower trough levels in females in a different series of our period.^7^, ^21^

For the surgery variables, as expected, fecal calprotectin levels were elevated before surgery. The most frequent reasons for surgery were stenosis or refractory disease in 49.1% and 22.8% of the cases, respectively, similar to other published studies.^22^, ^23^ Approximately 2/3 of the study population received infliximab and/or corticosteroids before surgery. Of the patients, 12.3% did not receive any medication before surgery, 19.3% had received only one medication, 24.6%, and 21.1% had two or three, and the remaining 22.9% had four or more medications, most of which were in the refractory disease category.

Few characteristic disparities were observed between the periods in this study. For the phenotyping of CD patients at diagnosis, there was a higher number of A1b patients in the 2019–2023 period (100%) in comparison to the earlier period (71.4%), but no significant disparities were observed in age at diagnosis before 2019 and after 2019 (median of 14.0 and 14.8 years, respectively). For medications received before surgery, there was a significant difference between patients who had received two and three medications. However, there was no difference between the two groups in the number of patients who received three or fewer medications.

This study still had its limitations as it was a single‐center study. The retrospective aspect of this study also limited the ability to identify the impact of the TDM approach between periods formally. Since 2019 marked the introduction of a standardized TDM approach at our center, this year was used as a cutoff with the expectation of demonstrating the effectiveness of the aimed infliximab trough levels at CHU Sainte‐Justine (15 µg/mL before 3rd dose and >10 µg/mL before 4th dose). However, it was impossible to compare both periods as the trough levels were measured too erratically before 2019. As suggested by Lujan et al.,^8^ we hypothesized that this could have decreased the incidence of surgeries.

## 5 CONCLUSION

To our knowledge, this study is among a small number of recent North American studies to report the incidence of surgery in pediatric IBD since the advent of earlier biologic initiation, demonstrating almost a threefold reduction in surgical rates compared to earlier years. Notably, our findings indicated a significant decrease in surgeries for newer cases of CD. While the phenotypes of patients undergoing surgery have remained consistent over the years, the early initiation of biologics was clearly a significant factor in the reduced necessity for surgical intervention since 2019. The improved proactive drug monitoring of infliximab may also have been a contributing factor, as therapeutic levels are now better established.^24^, ^25^, ^26^ Furthermore, the enhanced management of non‐responders and increasing use of small molecules in recent years will likely play a crucial role in the continued improvement of pediatric IBD treatment outcomes. Although larger cohorts are needed to corroborate these results, our findings represent promising and significant advancements in the pediatric IBD population.

## CONFLICT OF INTEREST STATEMENT

Laurence Chapuy received lecture fees from Janssen and AbbVie. Colette Deslandres is a speaker for Janssen and Organon. The remaining authors declare no conflicts of interest.

## References

[1] Coward S , Benchimol EI , Kuenzig ME , et al. The 2023 impact of inflammatory bowel disease in Canada: epidemiology of IBD. J Can Assoc Gastroenterol. 2023;6(suppl 2):S9‐S15. 37674492 10.1093/jcag/gwad004 PMC10478802

[2] Conrad MA , Kelsen JR . The treatment of pediatric inflammatory bowel disease with biologic therapies. Curr Gastroenterol Rep. 2020;22(8):36. 32542562 10.1007/s11894-020-00773-3 PMC8094805

[3] Agrawal M , Spencer EA , Colombel JF , Ungaro RC . Approach to the management of recently diagnosed inflammatory bowel disease patients: a user's guide for adult and pediatric gastroenterologists. Gastroenterology. 2021;161(1):47‐65. 33940007 10.1053/j.gastro.2021.04.063 PMC8640961

[4] Jenkinson PW , Plevris N , Siakavellas S , et al. Temporal trends in surgical resection rates and biologic prescribing in Crohn's disease: a population‐based cohort study. J Crohn's Colitis. 2020;14(9):1241‐1247. 32840295 10.1093/ecco-jcc/jjaa044

[5] Kugathasan S , Denson LA , Walters TD , et al. Prediction of complicated disease course for children newly diagnosed with Crohn's disease: a multicentre inception cohort study. Lancet. 2017;389(10080):1710‐1718. 28259484 10.1016/S0140-6736(17)30317-3 PMC5719489

[6] Walters TD , Kim MO , Denson LA , et al. Increased effectiveness of early therapy with anti‐tumor necrosis factor‐α vs an immunomodulator in children with Crohn's disease. Gastroenterology. 2014;146(2):383‐391. 24162032 10.1053/j.gastro.2013.10.027

[7] Sassine S , Djani L , Cambron‐Asselin C , et al. Risk factors of clinical relapses in pediatric luminal Crohn's disease: a retrospective cohort study. Am J Gastroenterol. 2022;117(4):637‐646. 35132979 10.14309/ajg.0000000000001650

[8] Lujan R , Buchuk R , Focht G , et al. Early initiation of biologics and disease outcomes in adults and children with inflammatory bowel diseases: results from the epidemiology group of the nationwide Israeli inflammatory bowel disease research nucleus cohort. Gastroenterology. 2024;166(5):815‐825.e22. 38331205 10.1053/j.gastro.2024.01.041

[9] Splawski JB , Pffefferkorn MD , Schaefer ME , et al. NASPGHAN clinical report on postoperative recurrence in pediatric Crohn disease. J Pediatr Gastroenterol Nutr. 2017;65(4):475‐486. 28937552 10.1097/MPG.0000000000001606

[10] Zhao M , Gönczi L , Lakatos PL , Burisch J . The burden of inflammatory bowel disease in Europe in 2020. J Crohn's Colitis. 2021;15(9):1573‐1587. 33582812 10.1093/ecco-jcc/jjab029

[11] Chaparro M , Garre A , Ricart E , et al. Differences between childhood‐ and adulthood‐onset inflammatory bowel disease: the CAROUSEL study from GETECCU. Aliment Pharmacol Ther. 2019;49(4):419‐428. 30637837 10.1111/apt.15114

[12] Jantchou P . Building a clinical and research database for children with inflammatory bowel disease (pedidata): a step‐by‐step process. J Pediatr Gastroenterol Hepatol Nutr. 2016;257:S81.

[13] Jakobsen C , Paerregaard A , Munkholm P , et al. Pediatric inflammatory bowel disease: increasing incidence, decreasing surgery rate, and compromised nutritional status: a prospective population‐based cohort study 2007–2009. Inflamm Bowel Dis. 2011;17(12):2541‐2550. 21381152 10.1002/ibd.21654

[14] Adamiak T , Walkiewicz‐Jedrzejczak D , Fish D , et al. Incidence, clinical characteristics, and natural history of pediatric IBD in Wisconsin: a population‐based epidemiological study. Inflamm Bowel Dis. 2013;19(6):1218‐1223. 23528339 10.1097/MIB.0b013e318280b13e PMC4898969

[15] Kolho KL , Nikkonen A , Merras‐Salmio L , Molander P . The need for surgery in pediatric patients with inflammatory bowel disease treated with biologicals. Int J Colorectal Dis. 2024;39(1):58. 38661931 10.1007/s00384-024-04634-7 PMC11045629

[16] Kayali S , Fantasia S , Gaiani F , Cavallaro LG , de'Angelis GL , Laghi L . NOD2 and Crohn's disease clinical practice: from epidemiology to diagnosis and therapy, rewired. Inflamm Bowel Dis. 2025;31(2):552‐562. 38582044 10.1093/ibd/izae075 PMC11808579

[17] Noor NM , Lee JC , Bond S , et al. A biomarker‐stratified comparison of top‐down versus accelerated step‐up treatment strategies for patients with newly diagnosed Crohn's disease (PROFILE): a multicentre, open‐label randomised controlled trial. Lancet Gastroenterol Hepatol. 2024;9(5):415‐427. 38402895 10.1016/S2468-1253(24)00034-7 PMC11001594

[18] Dhaliwal J , Walters TD , Mack DR , et al. Phenotypic variation in paediatric inflammatory bowel disease by age: a multicentre prospective inception cohort study of the Canadian children IBD network. J Crohn's Colitis. 2020;14(4):445‐454. 31136648 10.1093/ecco-jcc/jjz106 PMC7242003

[19] de Bie CI , Paerregaard A , Kolacek S , et al. Disease phenotype at diagnosis in pediatric Crohn's disease: 5‐year analyses of the EUROKIDS Registry. Inflamm Bowel Dis. 2013;19(2):378‐385. 22573581 10.1002/ibd.23008

[20] Rustgi SD , Kayal M , Shah SC . Sex‐based differences in inflammatory bowel diseases: a review. Therap Adv Gastroenterol. 2020;13:1756284820915043. 10.1177/1756284820915043 PMC7236567 32523620

[21] Girard C , Ackhar S , Sassine S , Chapuy L , Jantchou P , Deslandres C . A203 early proactive drug monitoring strategy of infliximab as monotherapy for inflammatory bowel disease in paediatric patients is associated with good sustained clinical remission. J Can Assoc Gastroenterol. 2023;6:44.

[22] Fumery M , Seksik P , Auzolle C , et al. Postoperative complications after ileocecal resection in Crohn's disease: a prospective study from the REMIND group. Am J Gastroenterol. 2017;112(2):337‐345. 27958285 10.1038/ajg.2016.541

[23] Gajendran M , Loganathan P , Catinella AP , Hashash JG . A comprehensive review and update on Crohn's disease. Dis Mon. 2018;64(2):20‐57. 28826742 10.1016/j.disamonth.2017.07.001

[24] Wilson A , Choi B , Sey M , Ponich T , Beaton M , Kim RB . High infliximab trough concentrations are associated with sustained histologic remission in inflammatory bowel disease: a prospective cohort study. BMC Gastroenterol. 2021;21(1):77. 33602145 10.1186/s12876-021-01650-7 PMC7890824

[25] Bevers N , Aliu A , Wong DR , et al. Early infliximab trough levels in paediatric IBD patients predict sustained remission. Therap Adv Gastroenterol. 2024;17:17562848231222337. 10.1177/17562848231222337 PMC10757796 38164362

[26] McDonald C , Kerr H , Gibbons E , et al. Higher ustekinumab levels in maintenance therapy are associated with greater mucosal healing and mucosal response in Crohn's disease: an experience of 2 IBD centers. Inflamm Bowel Dis. 2024;30(3):423‐428. 37158577 10.1093/ibd/izad073 PMC10906356

